# Minimally Invasive Approach to Treating Moderate Dental Fluorosis: A Case Report

**DOI:** 10.7759/cureus.74795

**Published:** 2024-11-29

**Authors:** Muhammad Mustaqim Nordin, Rosnani Mamat, Hafizah Ibrahim

**Affiliations:** 1 Conservative Dentistry and Endodontics, School of Dental Science, Universiti Sains Malaysia, Kota Bharu, MYS; 2 Conservative Dentistry, Universiti Sains Malaysia, Kota Bharu, MYS

**Keywords:** at-home whitening, dental fluorosis, hypomineralized enamel, minimally invasive intervention, resin infiltration

## Abstract

Dental fluorosis (DF) is a condition affecting tooth enamel that occurs during the development of permanent teeth, resulting from excessive fluoride consumption. Based on the severity, the tooth surface exhibits discoloration or structural anomalies. The range of colors varies from mild discoloration to severe dark brown lesions. These abnormalities lead to issues with appearance and impact the patient's self-esteem. This case report details a minimally invasive treatment for moderate DF in a 23-year-old male patient. The treatment involved a two-week at-home whitening regimen utilizing a 10% carbamide peroxide gel to diminish chromatic contrast on the affected teeth. Subsequently, enamel microabrasion was conducted to eliminate superficial discoloration prior to resin penetration on each maxillary tooth from UR5 to UL5. Following a two-year follow-up assessment, it was demonstrated that the tooth color was preserved, and no further discoloration occurred. This example showed that moderate DF can be addressed with a minimally invasive technique that integrates bleaching, microabrasion, and resin penetration.

## Introduction

Enamel hypomineralization is where the quality of enamel has been reduced. It occurs during the maturation stage of permanent teeth formation. Many factors could cause hypomineralization: local, systemic, environmental, and genetic factors. Dental fluorosis (DF) is a condition of enamel hypomineralisation caused by environmental factors. DF can be localized or generalized. The most common factor of generalized DF is due to long exposure to high fluoride concentrations in the water supply. This condition is noticeable in places or regions with high quantities of fluoride in their water sources [1]. DF can be identified through the discoloration on the tooth surface. A few indexes were used to categorize the DF severity. One of them is the Dean Fluorosis Index [2]. They ranged from normal to very severe. The aesthetic effects due to fluorosis are not a simple case. It is often given the negative impacts on patients' self-confidence and unmotivating them to face society. This issue will lead people to seek treatment to improve the appearance of their teeth.

Before this, the treatment for DF is more invasive such as veneers and crowns, which could result in significant loss of the more natural tooth structure. Nowadays, people look to minimally invasive treatment to achieve the treatment goal and preserve tooth integrity. For example, a previous study highlighted the success of minimally invasive treatment options such as home whitening, enamel microabrasion, and resin infiltration in the treatment of moderate fluorosis [3].

The present paper examines a 23-year-old male with moderate DF, who received treatment applying a combination of minimally invasive treatments. The treatment approach involved a 14-day at-home whitening procedure, proceeded by in-office microabrasion, and resin infiltration. Following a two-year follow-up assessment, the treated teeth exhibited no additional discoloration and maintained their original shade. A conservative treatment method for moderate DF effectively reaches sustainable aesthetic outcomes.

## Case presentation

**Figure 1:**
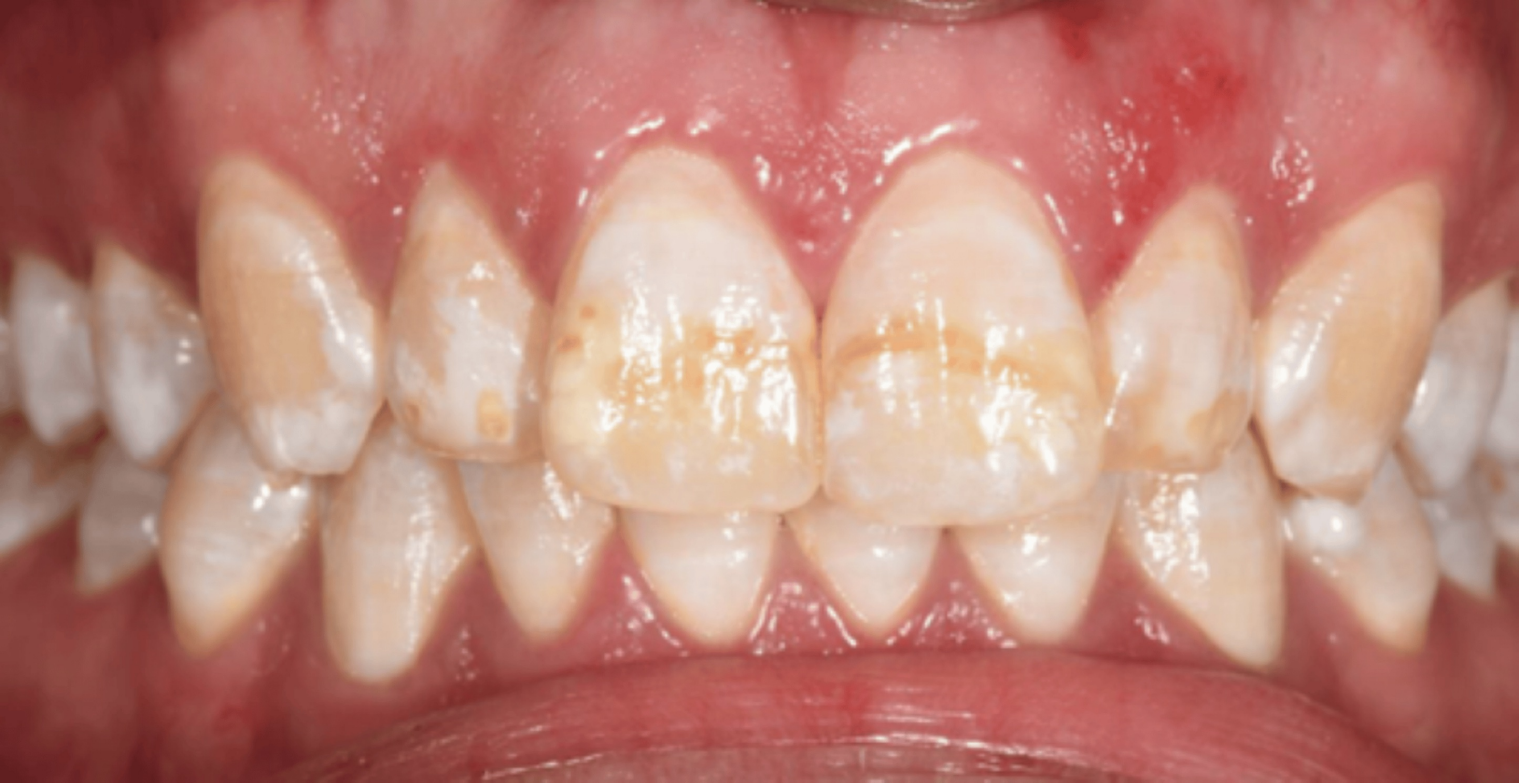
Clinical picture at initial presentation.

**Figure 2:**
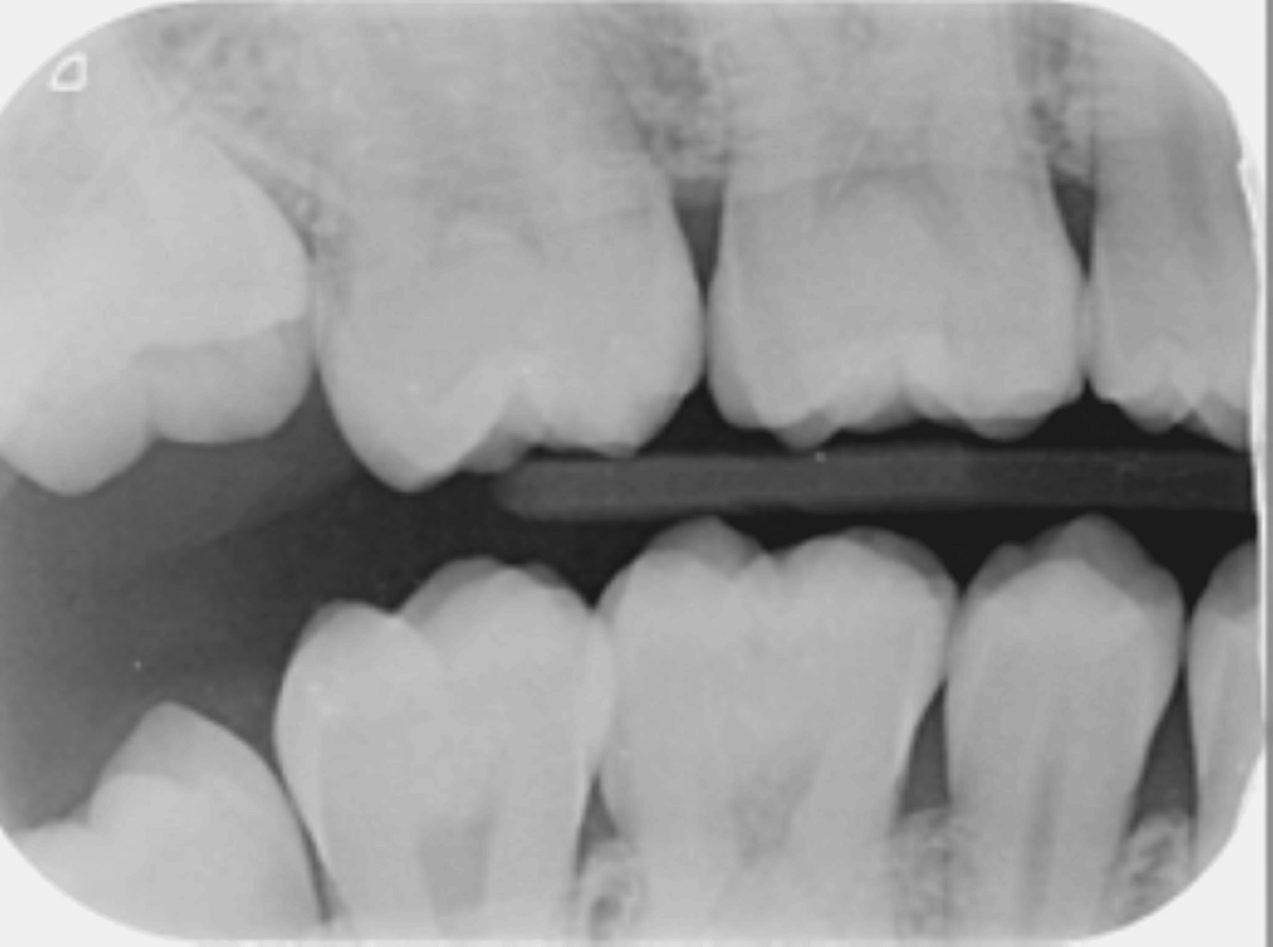
Bitewing radiograph of right quadrant.

**Figure 3:**
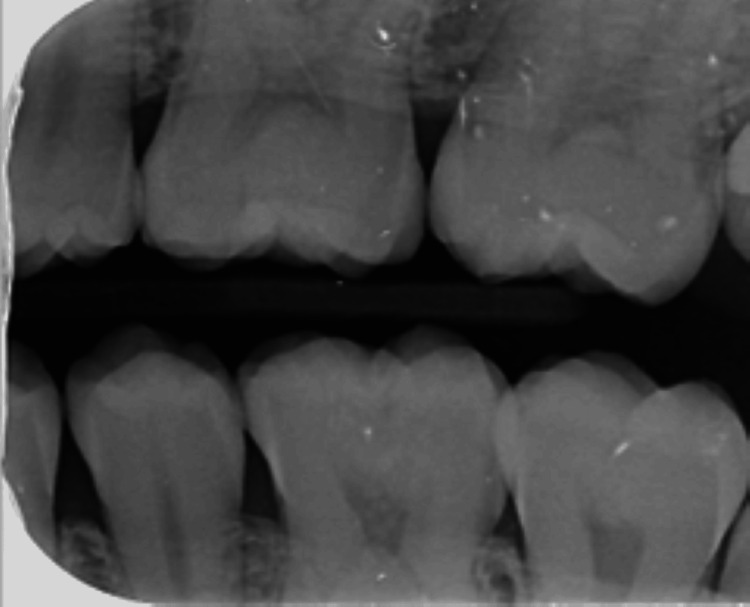
Bitewing radiograph of left quadrants.

A 23-year-old Yemeni male in good overall health was referred from the primary dental clinic to improve his inadequate aesthetic appearance. His primary concern was his upper front teeth, which exhibited uneven milky white and brown stains that significantly impacted his looks and smiles (Figure 1). He had never received treatment for this condition. He resided in Saada, Yemen, and maintained a healthy lifestyle. Intra-oral examination revealed his good oral condition. However, an inflammation was noticed on the buccal gingival area of tooth 22. Extra oral examination revealed normal findings, except for the unesthetic appearance when asked to make a smile. The radiographic assessment indicated that all teeth had a consistent periodontal ligament space and preserved lamina dura (Figures 2-3). The enamel surfaces are affected, with the brown stains on the anterior and posterior teeth supporting the dental fluorosis with a score of 4 according to the Dean Fluorosis Index [2] (Figure 1). The case was managed to improve the patient's aesthetic appearance as requested.

**Figure 4:**
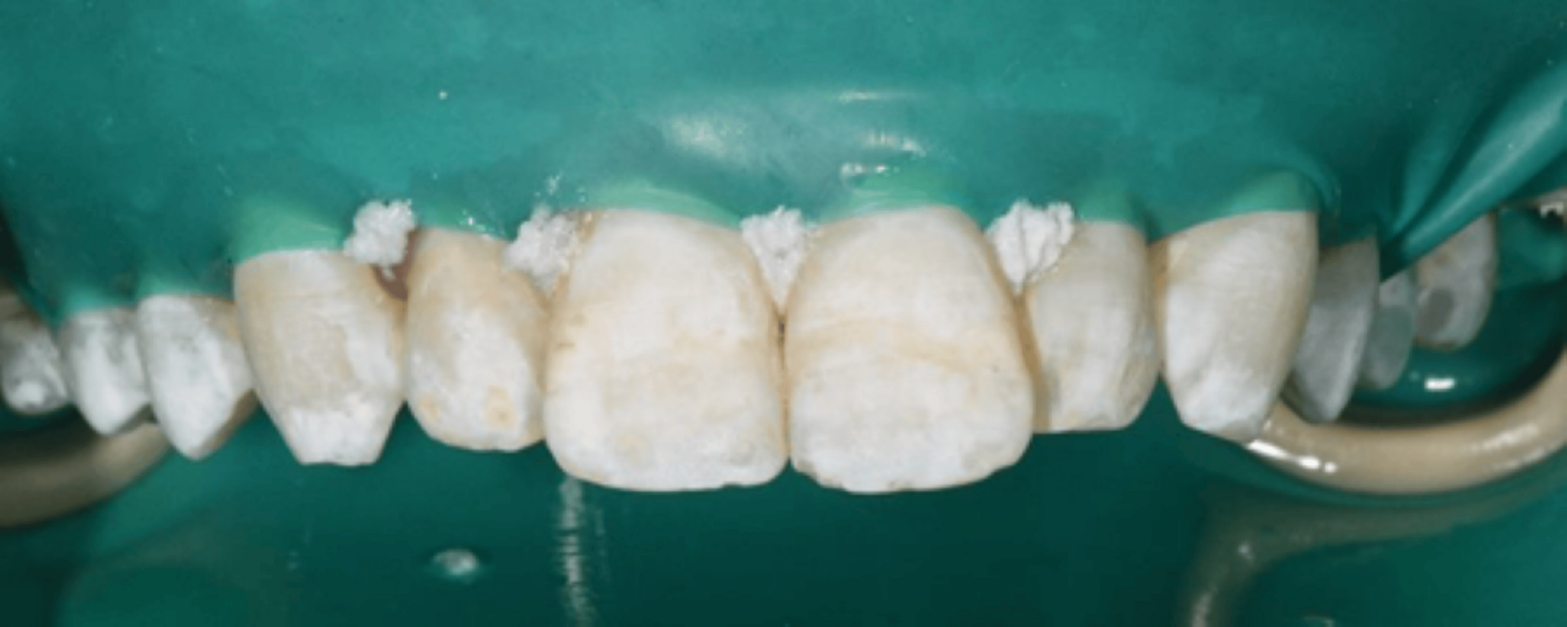
After completion of home bleaching for two weeks.

The proposed treatment plan for the patient involved home whitening utilizing a 10% carbamide peroxide gel for two weeks, applied for six to eight hours nightly, followed by enamel microabrasion with a 6.6% hydrochloric acid slurry and afterward resin infiltration treatment only for the maxillary arch, as requested by the patient. Upon the initial consultation, the patient provided consent for the treatment. Explanations and instructions about home whitening were given patient. An impression was obtained using alginate (Kromopan, Lascod, Italy) to create a working model, and custom arch trays were fabricated for at-home whitening. The patient utilized a nighttime maxilla treatment with 10% carbamide peroxide gel (Opalescence PF 10%, Ultradent Products, Inc.) for a duration of 14 days. However, he is not satisfied with his teeth’s appearance as there has been no improvement after the home whitening treatment. The photographs were captured subsequent to the completion of the home whitening procedure and prior to the initiation of the microabrasion treatment (Figure 4).

**Figure 5:**
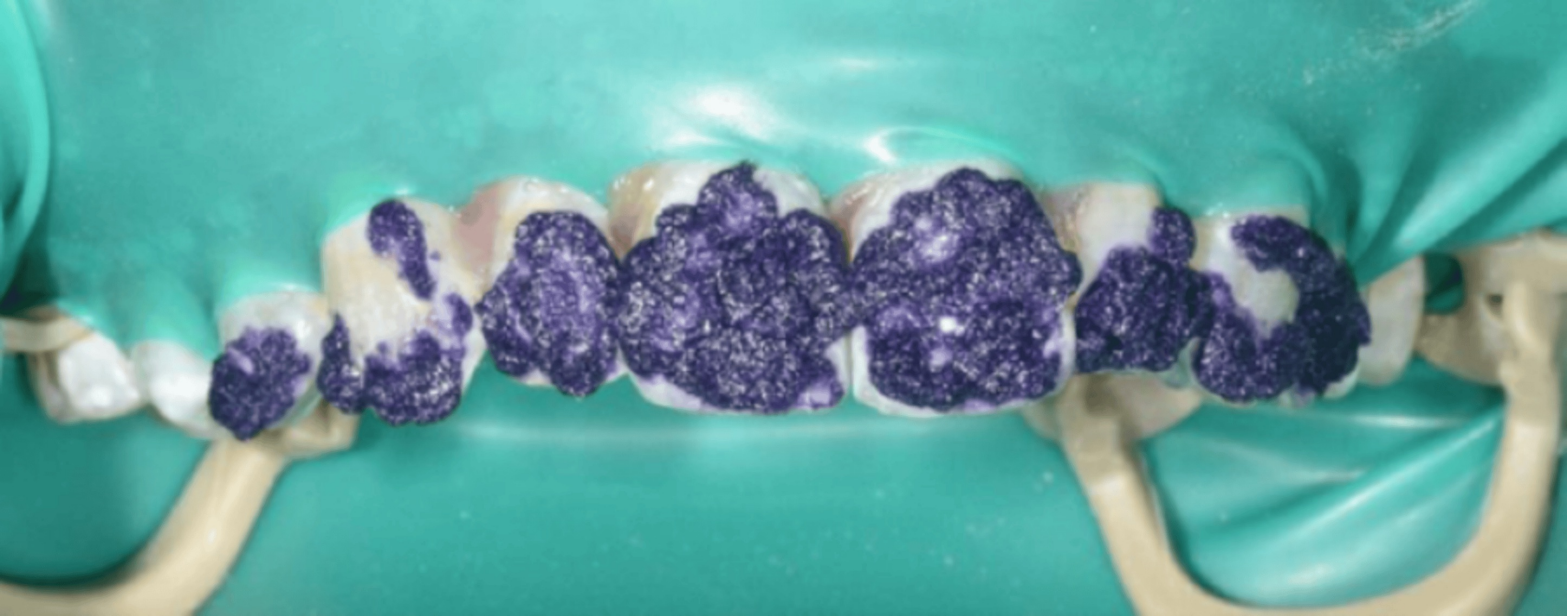
Opalustre 6.6% hydrochloric acid slurry was applied on the labial surface of the teeth.

**Figure 6:**
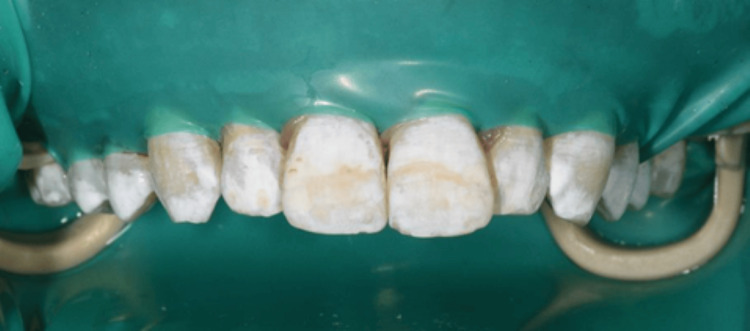
Post-microabrasion treatment. Some brown stains remained in the maxillary central incisors.

Rubber dam isolating was implemented on all addressed teeth (from UR6 to UL6) in order to protect the gingiva tissue. A mud combination (Opalustre, Ultradent Products, Inc., South Jordan, UT) was administered to the labial surfaces of the addressed teeth (Figure 5). A polishing cup (Opalcups, Ultradent Products, Inc.) was employed with a slow-speed contra-angle for one minute to eliminate excess mineralized enamel in preparation for tooth whitening. Frequent water rinses were conducted during the enamel microabrasion procedure. The procedure was executed twice (Figure 6).

**Figure 7:**
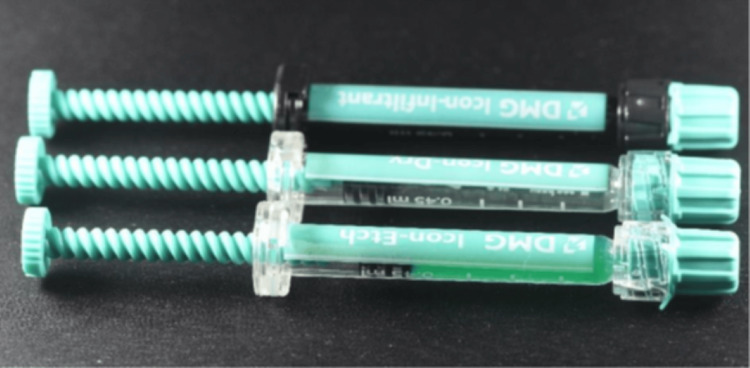
Resin infiltration system (Icon).

**Figure 8:**
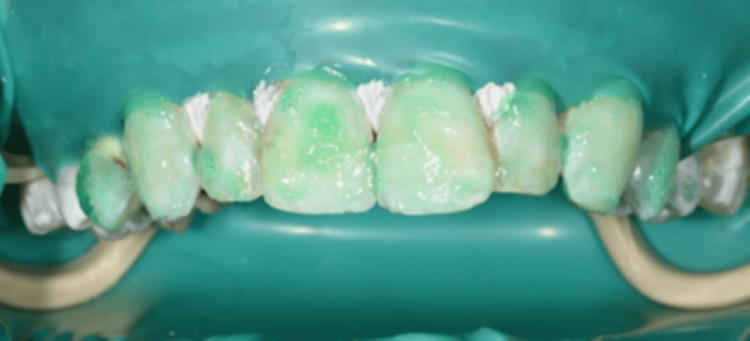
Icon-etch gel was applied to the maxillary teeth.

Treatment of resin infiltration started following the completion of the microabrasion procedure. A rubber dam was positioned before the start of treatment. A resin infiltration method (Icon DMG Chemisch-Pharmazeutische Fabrik GmbH, Hamburg, Germany) was utilized following the manufacturer's guidelines (Figure 7). The labial surfaces of this patient's teeth were etched three times for two minutes each using Icon-Etch gel, a 15% hydrochloric acid solution (Icon DMG Chemisch-Pharmazeutische Fabrik GmbH, Hamburg, Germany) (Figure 8). The teeth had been rinsed with water and had a typical matted appearance after acid etching.

**Figure 9:**
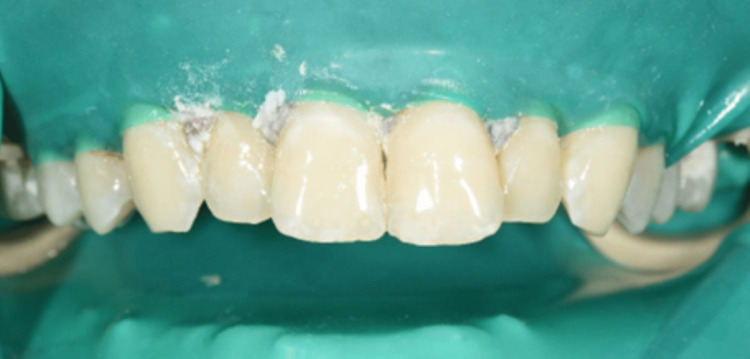
Icon-Dry was applied to the maxillary teeth.

Following rinsing and drying, the Icon-Dry solution (Icon DMG Chemisch-Pharmazeutische Fabrik GmbH, Hamburg, Germany), composed of 99% ethanol, was administered and allowed to act for 40 seconds to assess the expected end result (Figure 9). Upon mutual satisfaction of the preview phase by both the patient and physician, the subsequent step was to proceed.

**Figure 10:**
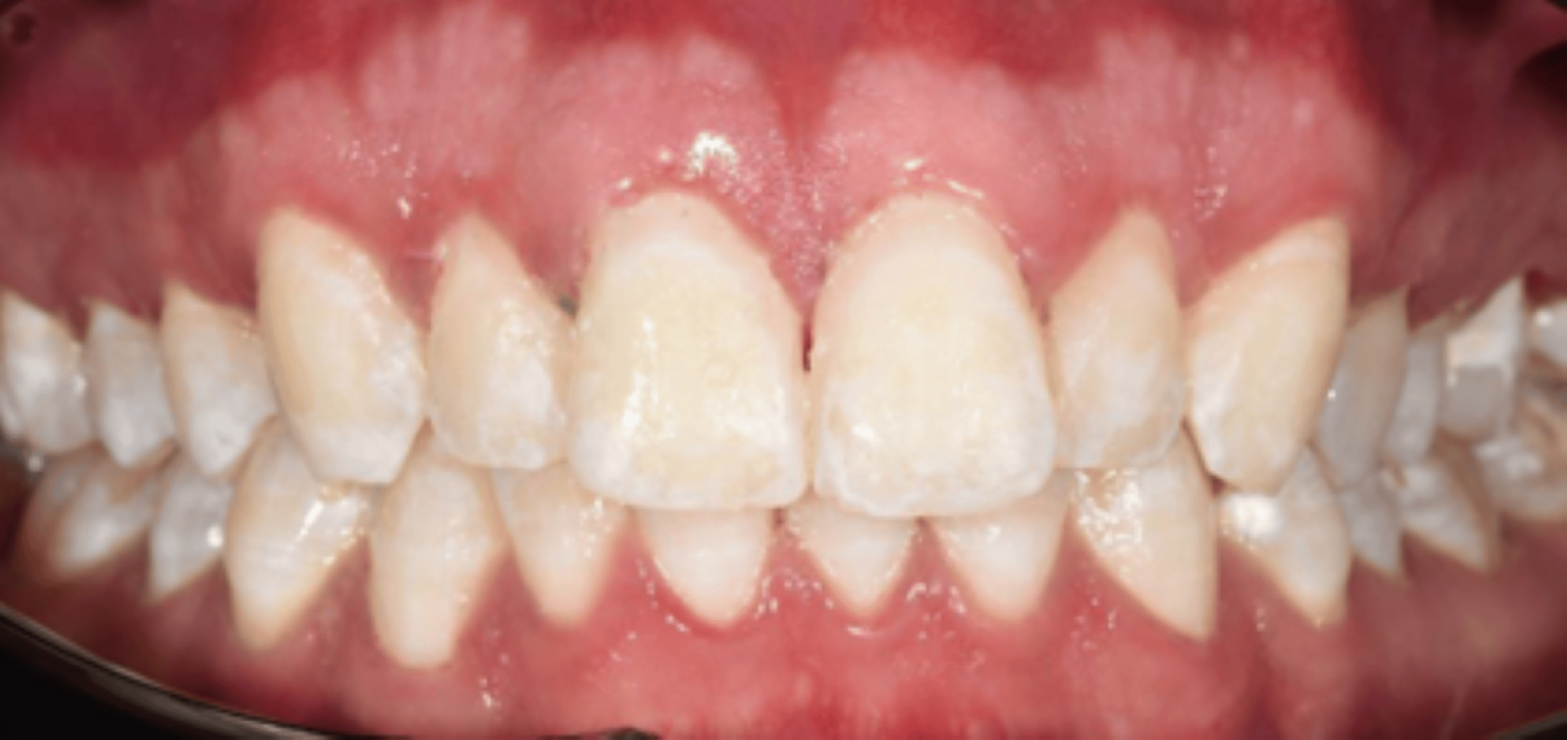
Immediately post-cure, the teeth surface is slightly rough due to excess resin.

Icon-Infiltrant (Icon DMG Chemisch-Pharmazeutische Fabrik GmbH, Hamburg, Germany) was administered to the affected tooth surfaces and allowed to remain for three minutes. The operational light was switched off to prevent early hardening of the resin. The residual resin was cleaned from the labial surface using a cotton pellet. The contacts were subsequently flossed to avoid adhesion between the teeth. Subsequently, cured with a light cure (VALO™ X; Ultradent Products Inc., South Jordan, UT) with an intensity of 1,100 mW/cm^2^ for 40 seconds. Immediately following the curing process, the Icon treatment results in a minor roughness of the teeth due to residual resin on the surfaces (Figure 10).

**Figure 11:**
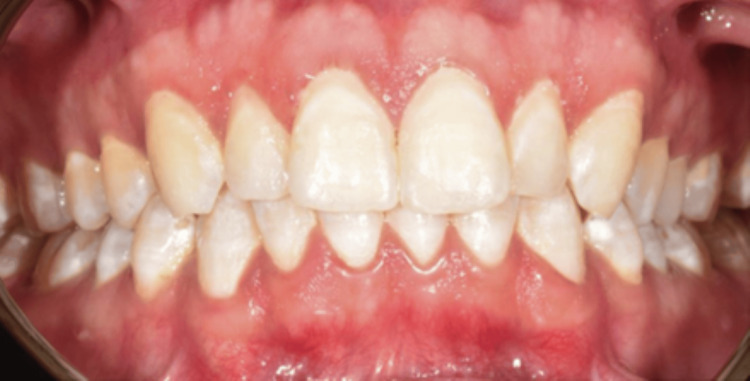
Post-operative image after two weeks.

**Figure 12:**
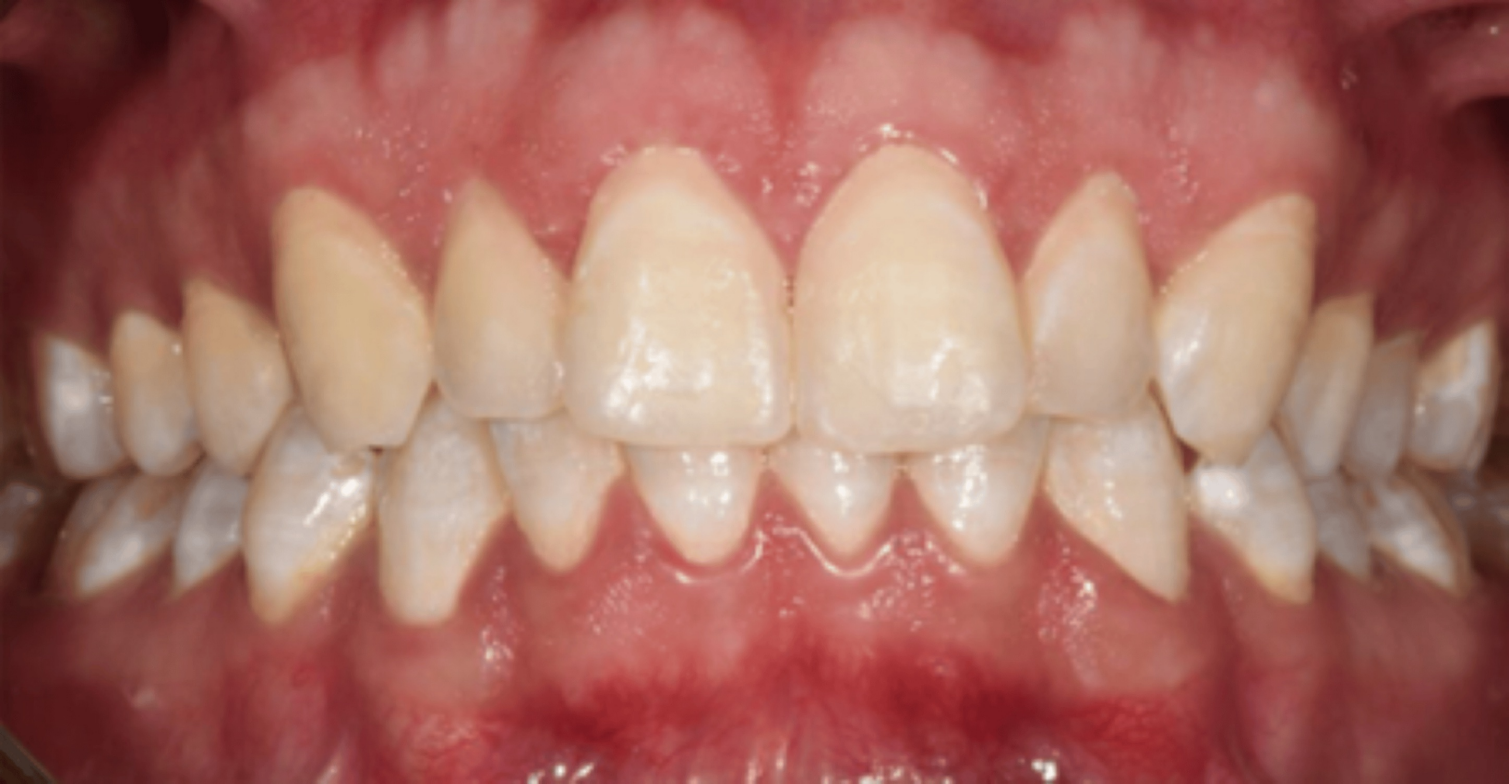
Two years post-operative resin infiltration.

Subsequent to the removal of the rubber dam, all treated teeth were polished using polishing cups to avoid external discoloration and enhance patient comfort. The patient had an examination after two weeks, and a postoperative image was captured. The patient expressed satisfaction with a notable aesthetic enhancement; the final DIF score was 0 (Figure 11). He was examined till the age of two years, during which the tooth color remained consistent. Oral hygiene was reinforced at each appointment (Figure 12).

## Discussion

In this case, the patient presented with generalized white spot lesions on his teeth since childhood. He was self-conscious about his appearance. The white spot lesion was diagnosed as DF. The treatment options suggested to the patient were in the sequence of the least invasive to the most invasive treatment approach: external bleaching, microabrasion, and resin infiltration.

One of the differential diagnoses of white spot lesions is hypomineralized enamel. Enamel is produced by ameloblast cells in two stages: the initial stage involves the secretion of matrix proteins, and the later stage is mineralization and maturation [4]. Hypomineralization of enamel is a disorder that happens during the mineralization or maturation process of enamel that caused reduce enamel quality. The enamel exhibits normal thickness but is inadequately mineralized, either presenting as opaque, creamy, or yellow or displaying brown discoloration [4]. These defects can be localized or generalized. Fluorosis can cause hypomineralization of tooth enamel, which can be either localized or generalized, characterized by diffuse, linear, or patchy white opacities lacking distinct borders.

Dental caries can also cause cloudiness or white spots on the surface of the teeth. Dental caries generally initiates at and beneath the enamel surface, resulting from the demineralization of crystalline minerals due to organic acids produced by bacteria. Prolonged acid exposure and pH reduction lead to an accelerated rate of mineral loss in the subsurface compared to the surface, culminating in the formation of a subsurface lesion that clinically manifests as a white spot due to increased porosity from mineral depletion [5]. The lesions are coded as International Caries Detection and Assessment System (ICDAS) codes 1 and 2 and are referred to as early-stage caries [6]. Changes in local ecology, dietary habits, and fluoride supply may cause the white spot lesion to stabilize and persist as an inactive lesion that does not advance, yet remains identifiable as a scar due to alterations in the optical characteristics of the enamel. The light is scattered by its irregular microstructure, and the result is an opaque white appearance of the tooth.

In this case, the patient denied any hospitalization, trauma, or previous orthodontic bracket placement, all of which may have contributed to enamel hypomineralization. Therefore, it was believed that the cause of the white spot lesions in this patient was due to excessive fluoride ingestion during childhood. The clinical appearance of the lesions consisted of symmetrical involvement of identical sets of teeth, cloudy calcified areas, and pinpoint puncture of the enamel with brown discoloration. This was supported by a patient born and raised in Saada, Yemen. According to Bagahizel, the prevalence of DF in Seda’a is 97.2% [7]. He also conducted a survey and found that 84% of people in Saada use tap water with an average concentration of 2.4 mg/L. While World Health Organization (WHO) recommended a maximum concentration of fluoride of 1.5 mg/L in drinking water as a safe and appropriate compromise between the chance of DF occurring and the protective effect against dental caries [1].

Recent studies indicate that a singular whitening treatment (e.g., microabrasion or home whitening) is beneficial solely for teeth exhibiting minor discoloration [8]. Achieving favorable outcomes in teeth affected by moderate to severe DF is challenging. In DF, there can be a hard, pathological layer of enamel on the tooth surface [9]. Therefore, as previously described, we started with home whitening treatment. Home bleaching had been performed to enhance the overall color of the teeth and thereby conceal the brown spots. Then, a microabrasion treatment with 6.6% hydrochloric acid was performed. The corrosive action of hydrochloric acid, together with the influence of silicon carbide particles, can eliminate around 25-200 micrometers of overmineralized enamel tissue [9]. The combination of home bleaching with 10% carbamide peroxide and microabrasion responded positively after two weeks. Nevertheless, the white spot lesions remained noticeable (Figure 6).

After home whitening, tooth color was allowed to stabilize for two weeks, and oxygen residues of tooth whitening were permitted to escape from the porous dental matrix. The risk of impeding the polymerization reaction of the resin in the resin infiltration system was simultaneously diminished. Capillary pressures facilitated the penetration of the low-viscosity infiltrant into the enamel lesions during infiltration. The refractive index of resin infiltration was similar to that of tooth enamel, which was 1.51 and 1.62, respectively [3]. Following the resin infiltration therapy, the patient's teeth exhibited a marked enhancement in their formerly chalky appearance. The patient was satisfied with the treatment result. He claimed that his confidence improved after treatment. To maintain good oral health, continuous monitoring of his oral hygiene every three months is necessary.

## Conclusions

This case study demonstrates that the integrated use of at-home whitening, microabrasion, and resin infiltration provides successful and aesthetic outcomes in managing moderate DF. The patient was delighted with the result of the treatment because there were huge differences between before and after treatment. Additionally, the approach is minimally invasive and cost-effective and aligns with conservative treatment principles. However, further studies on larger populations would be beneficial to validate these findings and establish standardized protocols for DF.
